# Association of Sleep Quality, Nutritional Factors, and Salivary Melatonin and Cortisol Levels with Oral Lichen Planus: A Case–Control Study

**DOI:** 10.3390/biomedicines14061275

**Published:** 2026-06-03

**Authors:** Éverton Adriano Wegner, Julia de Salles Teixeira, Gabriel Rübensam, Catieli Gobetti Lindholz, Fernanda Gonçalves Salum, Karen Cherubini

**Affiliations:** 1 Post-Graduate Program in Dentistry, School of Health and Life Sciences, Pontifical Catholic University of Rio Grande do Sul, Porto Alegre 90619-900, Brazil; everton.wegner@edu.pucrs.br (É.A.W.); fernanda.salum@pucrs.br (F.G.S.); 2 School of Health and Life Sciences, Pontifical Catholic University of Rio Grande do Sul, Porto Alegre 90619-900, Brazil; j.salles@edu.pucrs.br; 3 Toxicology and Pharmacology Research Center (INTOX), School of Health and Life Sciences, Pontifical Catholic University of Rio Grande do Sul, Porto Alegre 90619-900, Brazil; gabriel.rubensam@pucrs.br (G.R.); catieli.gobetti@pucrs.br (C.G.L.)

**Keywords:** oral lichen planus, sleep–wake disorders, melatonin, cortisol, diet

## Abstract

**Background/Objectives**: The etiology of oral lichen planus (OLP) is unknown, and the treatment is palliative. Considering the possible influence of factors related to lifestyle on the etiopathogenesis and behavior of OLP, the aim of the present study was to investigate the association of sleep quality, nutritional profile, and salivary melatonin and cortisol levels with OLP. **Methods**: Thirty-two OLP patients and 31 controls completed the Pittsburgh sleep quality index (PSQI), Epworth sleepiness scale, and 24 h dietary recall survey. Saliva was collected to determine melatonin and cortisol levels by liquid chromatography coupled to mass spectrometry. **Results**: The OLP patients showed higher scores in the sleep disturbances component of PSQI (*p* = 0.021) and lower salivary melatonin levels (*p* = 0.015), whereas salivary cortisol did not differ between the groups (*p* = 0.402). The Control group had higher prevalence of coffee drinkers (*p* = 0.045), whereas the OLP group had higher consumption of protein (*p* = 0.011), lipids (*p* = 0.043), calories (*p* = 0.022), monounsaturated fat (*p* = 0.030), polyunsaturated fat (*p* = 0.007), saturated fat (*p* = 0.027), cholesterol (*p* = 0.041), iron (*p* = 0.014), zinc (*p* = 0.048), magnesium (*p* = 0.025), sodium (*p* = 0.008), and vitamin E (*p* = 0.016) compared to controls. **Conclusions**: The results suggest that OLP is associated with lifestyle factors related to sleep and diet, as well as with lower levels of salivary melatonin. Given the exploratory nature of the study, further research is needed to better understand these findings.

## 1. Introduction

Oral lichen planus (OLP) is a chronic inflammatory immune-mediated disease where T lymphocytes have basal keratinocytes of the oral mucosa as a target [1]. OLP occurs more commonly in women from the fourth decade of life onward and has a worldwide prevalence of 1.01%. It differs in geographic distribution by region, with the highest prevalence in Europe and the lowest in India [2]. OLP has periods of relapse and remission, and according to clinical manifestations, it is classified into reticular, atrophic, erosive, papular, plaque-like, and bullous forms [1,2,3]. The disease has a chronic nature and can cause oral discomfort or even severe pain, feeding difficulties, sleep impairment, and psychological stress, especially in the erosive form. Such behavior significantly impairs the quality of life of patients [4].

The etiology of OLP remains unknown; however, environmental factors, genetic changes, oxidative stress, and metabolic dysfunction are believed to play a role by triggering immune system activation [1]. Additionally, psychological conditions that negatively impact quality of life, such as anxiety, depression, and chronic stress, appear to promote the onset of OLP and may also influence its clinical course [4,5,6]. That is, pre-existing psychological disorders can disturb the capacity to cope with the illness and pain perception, contributing to the severity of the cases [3]. The role played by stress would involve the activity of the hypothalamic–pituitary–adrenocortical axis (HPA), which has been investigated in individuals with OLP by quantifying cortisol, producing divergent results [7,8].

Studies investigating the relationship between OLP and sleep quality report that OLP patients seem to have worse sleep quality compared to the general population [9,10,11], where poor sleep has been claimed as a risk factor for the onset and aggravation of OLP [9]. However, until now, a few clinical studies have investigated such a relationship, most of them carried out in Europe. Some of these studies report OLP showing higher scores in the Pittsburgh sleep quality index (PSQI), but not in the Epworth sleepiness scale (ESS). Therefore, further research is needed, especially with other populations, to better understand this issue.

Melatonin (N-acetyl-5-methoxytryptamine), the major secretory product of the pineal gland [12], is the hormone most involved in regulating the oscillation between wakefulness and sleep [13]. Its secretion is controlled by the endogenous circadian cycle [14], decreasing with aging and being negatively correlated with poor sleep quality frequency [13]. The antioxidant effect is one of the major functions of melatonin [15], which also interacts in a complex way with the immune system [16]. Melatonin can have either anti-inflammatory or pro-inflammatory effects, depending on factors such as the type of cells involved and inflammation intensity and duration [16]. It was suggested that dysregulation in melatonin secretion would be associated with the pathogenesis of several autoimmune diseases, such as systemic lupus erythematosus, multiple sclerosis, and rheumatoid arthritis [17,18]. It is also believed that chronic inflammation in OLP could induce local melatonin biosynthesis, which would contribute to the clinical improvement of oral mucosal lesions [19].

Cortisol, the main stress hormone, and melatonin, the hormone regulating sleep, are both related to the circadian cycle and function in a coordinated and temporally opposing manner. Psychological stress, which has been associated with OLP, may indirectly inhibit melatonin activity, partly through increased cortisol secretion, leading to sleep disturbances. These hormones have been extensively analyzed in saliva. Salivary cortisol levels correlate with serum concentrations and reflect activation of the HPA axis, serving as an indicator of psychological stress [10]. Melatonin, in turn, is also released into saliva, where its levels reflect the proportion of freely circulating melatonin in plasma [12]. Moreover, saliva is an easily accessible biofluid that can be collected using non-invasive methods and has become an important source of biomarkers and a valuable diagnostic tool.

Finally, studies have suggested a link between metabolic syndrome and OLP [20]. A high-fat diet can amplify low-grade inflammation through infiltration of pro-inflammatory macrophages, oxidative stress, hypoxia, and lipolysis [21]. On the other hand, the consumption of products such as green tea has been reported as having therapeutic effects on OLP [22].

The etiology of OLP remains unclear, and treatment is primarily palliative, focusing on symptom control and the use of immunomodulatory therapies. Investigating factors potentially involved in the etiopathogenesis of the disease may contribute to a better understanding of its etiology and provide new perspectives for prevention and treatment. Sleep quality and diet significantly impact quality of life, while melatonin and cortisol serve as important biomarkers of circadian rhythm and stress-related physiological processes underlying these effects. Considering the possible influence of lifestyle-related factors on the etiopathogenesis and clinical behavior of OLP and the applicability of melatonin and cortisol as biomarkers in this context as well, the aim of the present study was to investigate the association of sleep quality, nutritional profile and salivary melatonin and cortisol levels with OLP.

## 2. Materials and Methods

### 2.1. Study Design

This work comprised a pilot observational case–control study, which was approved by the Research Ethics Committee of Pontifical Catholic University of Rio Grande do Sul (CEP-PUCRS) and was conducted in accordance with the ethical principles of the World Medical Association Declaration of Helsinki.

### 2.2. Participants

The sample consisted of 63 participants, 32 individuals with OLP and 31 control subjects. Participant recruitment took place from April 2022 to October 2023 and was based on convenience sampling, given the need to identify individuals meeting the inclusion and exclusion criteria, particularly the requirement for histopathological confirmation of OLP diagnosis. Eligible participants being seen at the Stomatology Outpatient Clinic were invited to participate in the study and signed a written informed consent. The Control group comprised individuals without oral diseases and was matched to the OLP group for age and sex. Male and female participants older than 18 years were included on the basis of the following inclusion/exclusion criteria. The inclusion criterion for the OLP group was a clinical diagnosis of OLP confirmed by histopathological examination [23], whereas the exclusion criterion specific to this group was a suspicion that the oral lesions were related to any drug use or dental restorative materials. The exclusion criteria for both groups were as follows: patients who underwent chemotherapy or radiotherapy; patients with immunodeficiency or autoimmune disease, pregnancy or breastfeeding; patients undergoing treatment with topical or systemic corticosteroids in the last 14 days or using melatonin supplements; and those unable to understand the questionnaires.

Sample size was calculated by using the GPower software (version 3.1.9.2) considering PSQI, ESS, and salivary melatonin, and based on Lopez-Jornet et al. [10], Cutando et al. [24], and Chang et al. [25]. It was estimated that 31 individuals would be needed per group, considering the *t*-test for independent samples, a 95% confidence level, two groups, and a power of 80%.

### 2.3. Procedures

At admission, information about age, sex, social habits (smoking, alcohol, coffee, and yerba mate use), weight, height, systemic diseases, and use of medication was recorded. The participants from both the OLP and Control group then underwent an intra- and extraoral examination. The questionnaires to assess sleep quality, insomnia, diet, and the presence and intensity of pain were administered after instructions on how to complete them were given.

In the OLP group, clinical assessment was carried out by using the scale developed by Thongprasom et al. [26]. The scale assigns the following score: 0 for normal mucosa with no lesions; 1 for mild white striae with no erythematous area; 2 for white striae with atrophic area less than 1 cm^2^; 3 for white striae with atrophic area more than 1 cm^2^; 4 for white striae with erosive area less than 1 cm^2^; and 5 for white striae with erosive area more than 1 cm^2^. The visual analog scale (VAS), ranging from 0 to 10, was used to assess the intensity of pain in the oral mucosa in OLP patients. The assessments were performed by a single examiner, with cases of uncertainty reviewed by a senior stomatologist.

The present study was reported in accordance with the Strengthening the Reporting of Observational Studies in Epidemiology (STROBE) guidelines for observational studies [27].

#### 2.3.1. Diet and BMI

Diet was assessed by a 24 h recall survey filled out three times on different days of the week, namely Sunday, Monday, and Wednesday. The nutritional profile analysis considered the following: macronutrients, calorie intake, lipid profile (fats) and micronutrients (minerals and vitamins). The Dietbox^®^ software (version 7.4.9) was used to analyze data where the daily intake of nutrients was determined. Weight and height were determined with participants barefoot and wearing light clothing, using a previously calibrated scale and stadiometer, respectively. Participants were positioned upright, relaxed, and with the head oriented with the Frankfurt plane parallel to the ground. Weight and height data were used to calculate the body mass index (BMI) [weight (kg)/height (m^2^)]. BMI was used as an indicator of nutritional status, since its combination with dietary intake data allows a more comprehensive characterization of the nutritional profile.

#### 2.3.2. Saliva Collection

Unstimulated saliva was collected in the morning, between 9 and 9:30 a.m., without fasting but at least 60 min after a meal and 12 h after drinking alcohol if used. Smokers were advised not to smoke for a minimum period of 60 min prior to collection; women were advised to avoid wearing lipstick. Before sample collection, the participants rinsed their mouth with water for 2 min, and saliva was then passively collected until obtaining 5 mL. The saliva was dispensed by the participant into a Falcon^®^ tube (Corning Inc., Corning, NY, USA) with the help of a funnel, and the date and time of collection were recorded. The collected saliva was kept on ice for at most 60 min while transported to the laboratory to be stored at −80 °C until analysis.

#### 2.3.3. Salivary Melatonin and Cortisol Levels

Prior to analysis, saliva samples were thawed at room temperature, protected from light, and then vortexed for 30 s. Next, 500 µL of the sample was mixed with 500 µL of acetonitrile with stirring at 70 rpm for about 5 min to precipitate protein. Centrifugation was performed at 4 °C, 14,000 rpm for 7 min, and the supernatant was transferred to polypropylene plates, stored at 4 °C, and protected from light until the extracts were analyzed by ultra-high-performance chromatography coupled with mass spectrometry (LC-MS/MS).

Measurement of melatonin and cortisol levels was carried out by LC-MS/MS, using an Infinity 1290 ultra-high-performance chromatograph (Agilent Technologies, Santa Clara, CA, USA), and a triple quadrupole mass spectrometer, Agilent 6460 (Agilent). The separation was carried out on a Zorbax Eclipse Plus Phenyl-Hexyl chromatographic column C18 (4.6 × 50 mm, 1.8 µm, Agilent, Santa Clara, CA, USA), and the mobile phases consisted of (A) 0.1% acetonitrile in ultrapure water and (B) 0.1% acetonitrile and 0.1% formic acid. The analytes were monitored in MRM mode for ions *m*/*z* 233 > 174 for quantification and confirmation of melatonin, and *m*/*z* 407 > 331 for quantification and confirmation of cortisol. Quantification was performed by external standardization, and the calibration curves were constructed in the range of 0.1 to 75 ng/mL. After analysis, the saliva samples collected were properly discarded.

#### 2.3.4. Sleep Quality

The validated Portuguese versions of the PSQI [28] and ESS [29] were used for subjective assessment of sleep quality. PSQI consists of 19 self-assessment questions and five questions that must be answered by the spouse or roommate. The last five questions are used for clinical information only. The 19 questions are categorized into seven components, classified as a score of 0 to 3. The PSQI components are subjective sleep quality, sleep latency, sleep duration, habitual sleep efficiency, sleep disturbances, use of sleeping medications, and daytime dysfunction. A global score is obtained by the sum of the scores for the seven components. This global score ranges from 0 to 21, where the higher the score, the worse the sleep quality.

The ESS is a self-administered questionnaire that assesses the probability of falling asleep in eight situations involving daily activities, some of which are known to be highly soporific. The score varies from 0 to 24, where values higher than 10 are indicative of excessive daytime sleepiness.

#### 2.3.5. Statistical Analysis

Data were analyzed by using descriptive and inferential statistics. Descriptive statistics included mean, standard deviation (SD), median, and interquartile range (IQR). The chi-square test was used to compare the frequency of the variables between the groups. Data normality was assessed using the Kolmogorov–Smirnov test with Lilliefors correction, and accordingly, the *t*-test for independent samples or the Mann–Whitney U test was used to compare the OLP and Control groups. Comparisons between the subgroups Erosive OLP and Non-erosive OLP and the Control were performed using the Kruskal–Wallis test. Correlations were tested with the Spearman correlation coefficient. SPSS software (IBM, Chicago, IL, USA, version 29) was used in the analysis at the level of significance of 5%. Effect size was calculated using Cliff’s Delta, an appropriate metric for nonparametric data. The interpretive criteria applied were as follows: absolute values < 0.15 indicate a negligible effect; values between 0.15 and <0.33 represent a small effect; values between 0.33 and <0.47 indicate a medium effect; and values ≥ 0.47 correspond to a large effect.

## 3. Results

### 3.1. Demographic Features and Habits of the Sample

The demographic characteristics and habits of the OLP patients and controls are presented in Table 1. The Control group showed a significantly higher prevalence of coffee drinkers (*p* = 0.045). Sex, age, frequency of smoking, and alcohol and yerba mate consumption did not significantly differ between the groups (*p* > 0.05). Data regarding the frequency of systemic diseases and regular use of medication in the OLP and Control group can be found in the Supplementary Material.

### 3.2. Sleep Quality

The results for sleep quality are summarized in Table 2. There was no significant difference for global PSQI (*p* = 0.105) and ESS (*p* = 0.126) scores between the OLP group and Control (Mann–Whitney U test, α = 0.05). In the PSQI components, however, the sleep disturbances score was significantly higher in the OLP group (*p* = 0.021), even though with a small effect size (Cliff’s Delta = 0.30; 95% CI = 0.04; 0.53). Subjective sleep quality (*p* = 0.481), sleep latency (*p* = 0.654), sleep duration (*p* = 0.061), habitual sleep efficiency (*p* = 0.057), use of sleep medications (*p* = 0.710), and daytime dysfunction (*p* = 0.839) did not significantly differ between the groups.

### 3.3. Salivary Melatonin and Cortisol Levels

**Figure 1:**
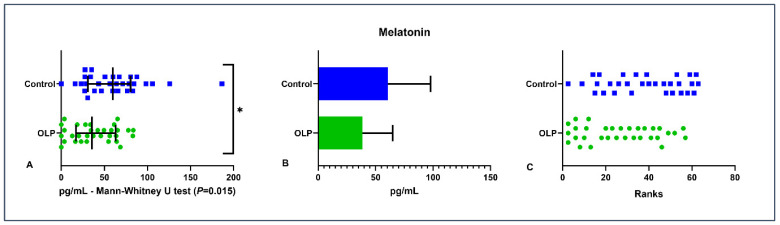
Salivary melatonin level in the OLP group (n = 32) and the Control group (n = 31). (**A**): median and IQR; (**B**): mean and SD; (**C**): ranks. * Mann–Whitney U test, *p* = 0.015. IQR = interquartile range; OLP = oral lichen planus; SD = standard deviation.

**Figure 2:**
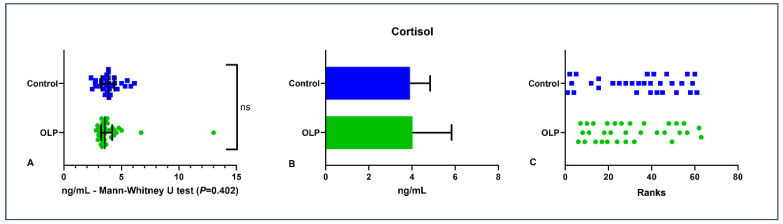
Salivary cortisol level in the OLP group (n = 32) and the Control group (n = 31). (**A**): median and IQR; (**B**): mean and SD; (**C**): ranks. ns = non-significant, Mann–Whitney U test, *p* = 0.402. IQR = interquartile range; OLP = oral lichen planus.

Salivary melatonin levels were significantly lower in the OLP group than in the Control (*p* = 0.015), with a medium size effect (Cliff’s Delta = −0.36; 95% CI = −0.59; −0.08), whereas salivary cortisol levels did not significantly differ between the groups (*p* = 0.402), with a negligible size effect (Cliff’s Delta= −0.12; 95% CI = −0.40; 0.17). These results are presented in Figure 1 and Figure 2.

### 3.4. Diet and BMI

BMI did not significantly differ between the OLP group and Control (*p* = 0.405; Mann–Whitney U test, α = 0.05). The OLP group showed a higher consumption of protein (*p* = 0.011), lipids (*p* = 0.043), and calories (*p* = 0.022) than controls. Also, in fat consumption analysis, values of monounsaturated fat (*p* = 0.030), polyunsaturated fat (*p* = 0.007), saturated fat (*p* = 0.027), and cholesterol (*p* = 0.041) were higher in the OLP group. Regarding electrolytes and minerals, OLP showed higher levels of iron (*p* = 0.014), zinc (*p* = 0.048), magnesium (*p* = 0.025), and sodium (*p* = 0.008). In addition, a higher vitamin E consumption was found in the OLP group (*p* = 0.016). The BMI and the dietary variables of the OLP patients and controls are presented in Table 3 (Mann–Whitney U test, α = 0.05). Considering the multiple nutritional variables analyzed, the results presented in Table 3 should be interpreted with caution. No adjustments for multiple testing were applied, and unadjusted *p*-values are reported alongside effect sizes and confidence intervals, providing a comprehensive assessment of the findings.

### 3.5. Analysis According to OLP Subgroup

**Figure 3:**
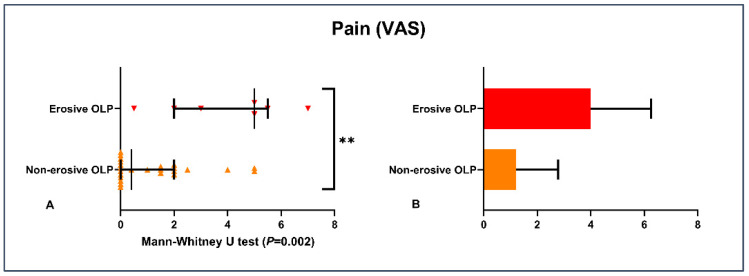
Pain intensity (VAS) in the Non-erosive OLP (n = 25) and Erosive OLP (n = 7) groups. (**A**): median and IQR; (**B**): mean and SD. ** *p* = 0.002. IQR = interquartile range; OLP = oral lichen planus; SD = standard deviation; VAS = visual analog scale.

**Figure 4:**
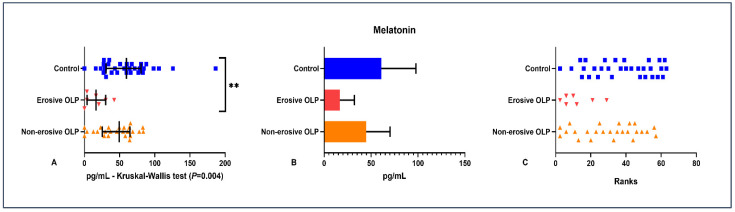
Salivary melatonin level in the Non-erosive OLP (n = 25), Erosive OLP (n = 7), and Control (n = 31) groups. (**A**): median and IQR; (**B**): mean and SD; (**C**): ranks. ** *p* = 0.004. IQR = interquartile range; OLP = oral lichen planus; SD = standard deviation.

**Figure 5:**
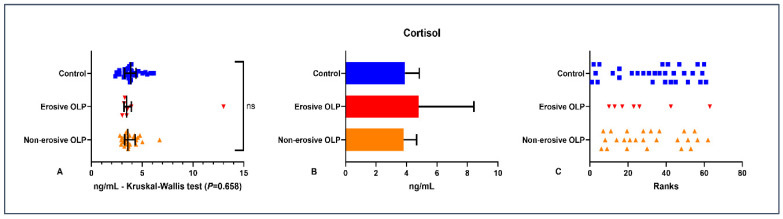
Salivary cortisol level in the Non-erosive OLP (n = 25), Erosive OLP (n = 7), and Control (n = 31) groups. (**A**): median and IQR; (**B**): mean and SD; (**C**): ranks. ns = non-significant, Kruskal–Wallis, *p* = 0.658. IQR = interquartile range; OLP = oral lichen planus; SD = standard deviation.

This section presents a complementary analysis grouping the OLP cases according to erosive and non-erosive classification. Most OLP cases were non-erosive (n = 25) and mainly affected the buccal mucosa. Among these, 7 cases were of reticular form, and 18 were atrophic. Erosive OLP corresponded to 7 cases and showed a significantly higher pain (VAS) score than Non-erosive OLP (*p* = 0.002) (Figure 3). Comparisons between the Non-erosive OLP (1, 2 and 3 Thongprasom scores), Erosive OLP (4 and 5 Thongprasom scores), and Control groups are presented in Figure 4 and Figure 5 and Table 4. Salivary melatonin level was highest in the Control group, followed by the Non-erosive OLP and Erosive OLP levels, with the latter OLP level being significantly lower compared to the Control group (*p* = 0.004). Salivary cortisol levels did not significantly differ between the groups (*p* = 0.658). In PSQI (Table 4), the use of sleep medications was higher in the Erosive OLP group, followed by Control and by Non-erosive OLP, with a significant difference occurring between Erosive OLP and Non-erosive OLP (*p* = 0.040).

### 3.6. Correlations

When correlations between PSQI (global), EES, BMI, melatonin, and cortisol were examined, a weak negative correlation between cortisol and PSQI was observed at the 0.05 significance level. No other significant correlations were found between these variables (Spearman correlation coefficient, Table 5).

## 4. Discussion

This work investigated the relationship of sleep quality, nutritional factors, and salivary melatonin and cortisol levels with OLP. Sleep is crucial for quality of life, so much so that sleep deprivation exerts severe damage to health [9]. We found a tendency for higher scores in the global PSQI and ESS in OLP patients, especially in Erosive OLP. However, the difference between them and controls was not significant. In the same way, previous studies have found no difference between OLP patients and controls in the ESS [9,10,11]. However, when analyzing PSQI components, we found a significantly higher score for sleep disturbances in the OLP group, which agrees with a previous report [9]. Also, the use of the sleep medication component was significantly higher in the Erosive OLP subgroup. Still, corroborating such a notion, we found melatonin, considered a sleep hormone, at lower levels in OLP patients, and even lower in Erosive OLP. These findings suggest that OLP patients could have worse sleep quality than the general population, corroborating Adamo et al. [11]. The possibility of poor sleep playing a role in OLP etiopathogenesis would be supported by the deregulation effects it exerts on the immune system, especially increasing pro-inflammatory cytokine production. Meanwhile, it has been reported that melatonin reduces inflammation in patients with insomnia [30].

Regarding the lower melatonin levels found in OLP, some additional comments would be appropriate. This chronobiotic pineal gland hormone [16] is also produced at extra-pineal sites [31,32] and has anti-inflammatory and pro-inflammatory effects [16]. Melatonin works as an immunostimulant, antagonizing the immunosuppressive effects of cortisol and stimulating lymphocyte activity [31]. It also has antioxidant effects [15]. Considering these properties of melatonin, we can infer that the lower levels found in the OLP group could contribute either systemically or locally to the development and aggravation of OLP lesions, in agreement with reports on the involvement of melatonin in the pathogenesis of autoimmune diseases [17,18]. Conversely, Luengtrakoon et al. [32] reported greater melatonin levels in mucosal samples of OLP biopsies. According to the authors, this suggests that chronic inflammation in OLP demands local biosynthesis of melatonin as a physiological demand for cytoprotection.

A theory of involvement of neuroendocrine responses to stress in the etiopathogenesis of OLP has been proposed [19]. Cortisol, a biomarker of anxiety and stress [33], has been analyzed in OLP patients with divergent results [7,8,10]. In our study, no significant difference in salivary cortisol levels was observed between OLP patients and controls, consistent with previous reports [8,34,35]. Lopez-Jornet et al. [10], on the other hand, found significantly higher levels of salivary cortisol in individuals with OLP than in controls. Similarly to our study, these authors collected unstimulated saliva in the morning. However, while their collections were between 10:00 am and 12:00 am, ours were between 9:00 am and 9:30 am. The method of cortisol assessment also differed between the studies, but we do not know whether this would be related to the discrepancy in the results. Considering the circadian fluctuations in cortisol levels, repeated assessments at different times throughout the day would provide more comprehensive insights. The weak negative correlation observed between cortisol and PSQI supports this notion, suggesting that individuals with poorer sleep quality may exhibit lower cortisol levels upon awakening. However, a larger sample size could provide more precise estimates of the correlations between these and the other variables in the study.

A meta-analysis found higher salivary cortisol levels in OLP patients only in India, and by using an ELISA method. In studies conducted in the Middle East, Europe, and Brazil, or using other methods (radioimmunoassay, chemiluminescent immunoassay), there was no significant difference between OLP patients and controls [36].

In our study, OLP patients’ diet showed higher consumption of protein, lipids, sodium, calories, cholesterol, and monounsaturated, polyunsaturated, and saturated fats than that of controls. These are known as characteristics of the Western diet, which has been linked to etiopathogenesis of autoimmune diseases, where the involvement of adipocytokines, dysbiosis [37], and impaired intestinal barrier function has been discussed [38]. Obese and hyperlipidemic individuals are prone to develop a variety of chronic diseases, not only autoimmune ones, with some evidence of the involvement of pro-inflammatory cytokine secretion [37]. It seems that cytokines secreted by hypertrophic adipocytes lead to low-grade inflammation amplified by pro-inflammatory factors [21]. Accordingly, OLP patients are more susceptible to developing metabolic syndrome [20], and clinical studies have shown an association between OLP and dyslipidemia [39,40]. Although our OLP group showed a BMI indicative of overweight [41], there was still no significant difference compared to controls. In agreement with this result, the literature reports that dietary saturated fatty acids amplify skin inflammation in psoriasis independently of obesity factors [21]. Recent evidence has linked a high-fat diet, dyslipidemia, and obesity to OLP progression. Furthermore, increased consumption of processed foods and dietary fats may contribute to a higher risk of OLP development [42].

We found higher intake of zinc, iron, magnesium, and vitamin E in the OLP group, which was at first unexpected, since these compounds are recognized as having protective effects on tissues. Zinc is essential for epithelium growth [43] and for the normal function of immune cells [44]. Its deficiency impairs wound healing and induces T-helper 2 cell cytotoxicity [43]. However, excessive zinc can impair the immune system comparably to its deficiency. Healthy zinc homeostasis is essential in both protecting the human body from pathogens and preventing overreaction of the immune system. Dietary iron and magnesium, in turn, have already been positively associated with the risk of metabolic syndrome [45]. Regarding the vitamins, it is known that they play an important role in regenerative processes [46], while their deficiency causes susceptibility to systemic and oral diseases, with well-recognized manifestations in the oral mucosa [47]. Vitamin E particularly inhibits lipid peroxidation of the cell membrane [46]. A meta-analysis by Wang et al. [48] found no significant difference in salivary vitamin E levels between OLP patients and controls. Differently, our OLP patients showed higher consumption of vitamin E, even though the values were below the intake recommendation [49]. The vitamin E finding seems reasonable considering the higher intake of fats observed in the OLP group [50,51].

In fact, an analysis of dietary patterns should take into account the combined elements of the diet and not only their isolated behavior. Our findings of OLP subjects having a diet comprising higher intake of some nutrients support a predominant Western diet pattern in this group, which has been related to immune-mediated diseases [38]. On the other hand, the Mediterranean diet, a well-known healthy dietary pattern, besides being based on fruits, vegetables, and legumes, also includes coffee consumption [52], which is in agreement with our finding of higher prevalence of coffee drinkers in the Control group. In this regard, the literature has addressed the therapeutic effects of plant-derived preparations, including coffee, where they could interfere with different biochemical pathways, exerting antioxidant, analgesic, and anti-inflammatory effects [53]. Moreover, salivary IL-1α concentrations have been reported to be lower in caffeine consumers [54], whereas elevated levels of this interleukin are associated with Erosive OLP [55].

Our pain VAS analysis showed that both Erosive OLP and Non-Erosive OLP subjects reported oral pain at different intensities. However, for Erosive OLP, pain frequency and intensity were higher, which seems obvious and is also in line with the literature [10,11,56]. In agreement with the fact that persistent pain is an important cause of sleep disturbances in older adults [57], it is plausible that symptomatic OLP could impair the quality of sleep of the patients [11]. Otherwise, a bidirectional relationship may also exist between sleep quality and pain. That is, pain interferes with the ability to initiate and maintain sleep, and meanwhile, the persistence of untreated poor sleep can intensify the perception of pain [58].

Even though applying strict inclusion and exclusion criteria and sex- and age-matching of the groups, this was a clinical observational study with the limitations inherent in such a design. The convenience and small sample size, especially in the subgroup analysis, the absence of assessment of anxiety and depression, and the possible effect of some confounding factors are limitations to be acknowledged. Still, one may point out the risk of false-positive findings due to the large number of variables analyzed without correction for multiple comparisons. Although methods used to adjust *p*-values reduce the risk of type I errors, they may also decrease statistical power and increase the likelihood of false negatives. Therefore, we presented unadjusted *p*-values alongside effect sizes and confidence intervals [59,60]. In this context, the results should be considered exploratory and cautiously interpreted. Also, assessment of salivary melatonin and cortisol was carried out only once a day. Moreover, the questionnaires used to assess sleep and diet are susceptible to biases such as individual subjectivity of the respondents. Anyway, a plethora of methodological details were respected to minimize biases. To measure both melatonin and cortisol, we used liquid chromatography coupled to mass spectrometry, a highly accurate technique and a powerful tool for providing precise results. Regarding the questionnaires used, the 24 h dietary recall is considered a valid instrument for assessing energy and nutrient intake, being the most thorough, comprehensive, and complete. Nevertheless, it lacks representativity if applied only once and depends on the interviewee’s memory [61]. To reduce these biases, the 24 h dietary recall survey was conducted on three different days of the week. Furthermore, according to Salvador Castell et al. [62], this survey tends to underestimate food intake, especially in older people. OLP occurs at an older age, making it unlikely to carry out this type of study in a younger population. To reduce this bias, OLP patients were matched by age with controls. Another limitation here is that a 24 h dietary recall cannot represent long-term habitual dietary intake of participants, which could bring measurement bias. However, considering that our aim was the comparison of diet between the groups, we believe that our objective was well accomplished, since the same instrument was applied to all participants following the same criteria. Still, regarding the methods applied, sleep and dietary intake data collection was based on instruments where the subjectivity of the respondents could interfere with the results. In this scenario, examinations such as polysomnography and biochemical methods for assessing nutrients would be of help.

Up to now, this is one of the few investigations assessing dietary patterns, and to our knowledge, it is the first to evaluate sleep, including salivary melatonin levels in OLP patients. On the basis of the present study, it is possible to suggest that OLP is associated with particular aspects of both sleep quality and dietary pattern and with lower salivary melatonin levels as well. The association could be either indicative of these variables playing a role in the etiopathogenesis of OLP or just a consequence of a chronic disease that causes periods of pain or discomfort, thereby affecting the nutritional status as well as the quality of life of the patients [63]. The findings highlight the need for future research, including case–control studies with larger sample sizes, more specific and robust assessment methods, and proper control of potential biases to confirm the associations. Longitudinal and interventional studies focusing on the potential effects of dietary modulation, stress management, and melatonin administration on the clinical behavior of OLP would be of help to investigate causal relationships. In addition, further studies addressing stress, anxiety, and depression, along with cortisol and melatonin levels, are warranted to better elucidate the interaction of these conditions and OLP.

## 5. Conclusions

The results suggest that OLP is associated with lifestyle factors related to sleep and diet, as well as with lower levels of salivary melatonin. Given the exploratory nature of the study, further research is needed to better understand these findings.

## Figures and Tables

**Table 1:** Demographic features and habits of OLP patients and controls.

Features	OLP (n = 32)		Control (n = 31)		*p*
	n	%	n	%	0.707 *
Male	6	18.8	7	22.6	
Female	26	81.3	24	77.4	
Age (years)	Mean	SD	Mean	SD	0.260 **
	61.1	9.2	63.9	10.2	
Habits					
Smoking	n	%	n	%	0.719 *
Yes	1	3.1	2	6.5	
No	15	46.9	16	51.6	
Former smoker ^#^	16	50	13	41.9	
Alcohol	n	%	n	%	0.197 *
Yes	0	0	1	3.2	
No	32	100	28	90.3	
Former drinker ^##^	0	0	2	6.5	
Coffee	n	%	n	%	0.045 *
Yes	21	65.6	27	87.1	
No	11	34.4	4	12.9	
Mate	n	%	n	%	0.719 *
Yes	12	37.5	13	41.9	
No	20	62.5	18	58.1	

* Chi-square test; ** *t*-test for independent samples; significant if *p* ≤ 0.05; ^#^ former smokers (abstinent ≥ 6 months); ^##^ former drinkers (abstinent ≥ 6 months); OLP = oral lichen planus; SD = standard deviation.

**Table 2:** Sleep quality parameters in the OLP group and the Control group.

Sleep Parameters	OLP (n = 32)				Control (n = 31)				*p* *	Cliff’s Delta ** (95% CI)
	Median	IQR	Mean	SD	Median	IQR	Mean	SD		
PSQI (global)	9.0	6	8.75	4.158	6.0	6	7.23	4.417	0.105	0.24 (−0.06; 0.50)
PSQI (components)										
Subjective sleep quality	1	1	1.34	0.787	1	1	1.23	0.805	0.481	0.09 (−0.17; 0.34)
Sleep latency	2	1	1.56	1.045	1	2	1.45	1.121	0.654	0.06 (−0.22; 0.33)
Sleep duration	1.5	2	1.34	1.096	0	1	0.84	1.068	0.061	0.26 (−0.02; 0.50)
Habitual sleep efficiency	1	2	1.28	1.054	0	1	0.84	1.186	0.057	0.26(−0.02; 0.51)
Sleep disturbances	2	1	1.75	0.718	1	1	1.42	0.502	0.021	0.30 (0.04; 0.53)
Use of sleep medications	0	1	0.72	1.198	0	2	0.68	1.194	0.710	0.04 (−0.19; 0.27)
Daytime dysfunction	1	1	0.75	0.718	1	1	0.77	0.669	0.839	−0.03 (−0.29; 0.24)
ESS	6.5	7	7.53	4.189	6.0	6	5.87	4.602	0.126	0.22 (−0.07; 0.48)

* *p* value for Mann–Whitney U test; significant if *p* ≤ 0.05; ** absolute values < 0.15 indicate a negligible effect; values between 0.15 and <0.33 represent a small effect; values between 0.33 and <0.47 indicate a medium effect; and values ≥ 0.47 correspond to a large effect. ESS = Epworth sleepiness scale; IQR = interquartile range; OLP = oral lichen planus; PSQI = Pittsburgh sleep quality index; SD = standard deviation.

**Table 3:** BMI and dietary variables in the OLP group and the Control group.

	OLP (n = 32)				Control (n = 31)					
	Median	IQR	Mean	SD	Median	IQR	Mean	SD	*p* *	Cliff’s Delta ** (95% CI)
BMI	28.11	6.82	28.37	4.28	27.70	5.63	27.49	5.21	0.405	0.13 (−0.16; 0.40)
Dietary variables										
Macronutrients										
Protein (g)	79.31	38.30	85.75	28.96	63.68	30.65	67.54	29.06	0.011	0.37 (0.08; 0.60)
Carbohydrate (g)	169.71	83.83	183.49	63.53	177.18	80.97	196.85	131.17	0.945	0.01 (−0.27; 0.29)
Lipid (g)	57.11	37.96	63.08	29.99	46.64	27.84	48.00	17.41	0.043	0.30 (0.01; 0.55)
Sugar (g)	34.98	28.53	39.72	25.80	34.13	34.86	44.29	30.64	0.805	−0.04 (−0.32; 0.25)
Fibers (g)	15.48	8.33	17.03	6.78	12.64	12.38	14.95	7.12	0.165	0.20 (−0.09; 0.47)
Calories	1695.16	626.08	1661.95	474.35	1402.00	529.67	1387.99	398.41	0.022	0.33 (0.04; 0.57)
Fats										
Monounsaturated fat (g)	17.92	17.02	21.45	12.89	14.84	6.85	14.99	6.73	0.030	0.32 (0.03; 0.56)
Polyunsaturated fat (g)	11.53	10.19	12.94	7.08	7.77	6.21	8.10	4.54	0.007	0.40 (0.11; 0.62)
Saturated fat (g)	20.97	13.59	21.80	9.51	15.88	7.01	16.37	6.66	0.027	0.29 (0.00; 0.54)
Trans fat (g)	1.00	1.36	1.36	1.12	1.02	0.89	1.33	1.58	0.747	0.05 (−0.24; 0.33)
Cholesterol (mg)	279.52	194.86	315.32	184.87	199.00	185.43	236.88	156.53	0.041	0.30 (0.00; 0.55)
Electrolytes and minerals										
Calcium (mg)	458.36	224.96	478.02	189.29	396.78	302.49	218.65	39.27	0.182	0.20 (−010; 0.46)
Iron (mg)	12.30	4.62	12.05	3.78	9.61	3.92	9.48	3.58	0.014	0.36 (0.07; 0.60)
Zinc (mg)	10.16	4.31	11.17	5.08	8.53	3.01	9.04	3.74	0.048	0.29 (−0.00; 0.54)
Magnesium (mg)	198.66	81.99	216.82	62.85	170.09	122.43	178.34	73.80	0.025	0.33 (0.03; 0.57)
Sodium (mg)	1571.25	1074.20	1696.39	807.53	1037.58	976.18	1166.88	636.03	0.008	0.39 (0.10; 0.61)
Phosphorus (mg)	873.25	461.51	953.21	327.59	785.98	413.34	778.67	304.89	0.065	0.27 (−0.02; 0.52)
Potassium (mg)	2123.45	944.73	2268.44	639.48	1734.12	1239.65	1942.82	785.06	0.058	0.28 (−0.02; 0.53)
Selenium (µg)	86.02	55.10	102.31	53.07	80.79	35.59	80.81	39.23	0.102	0.24 (−0.05; 0.49)
Manganese (mg)	1.82	1.13	2.26	1.41	1.65	1.31	1.80	1.07	0.073	0.24 (−0.06; 0.50)
Vitamins										
A (µg)	255.55	256.90	293.91	174.87	255.16	328.34	342.92	330.90	0.721	0.03 (−0.26; 0.31)
B1 (mg)	1.11	0.50	1.26	0.66	0.99	0.77	1.10	0.49	0.296	0.15 (−0.14; 0.42)
B12 (µg)	3.27	2.87	4.19	2.68	2.91	1.22	3.06	1.80	0.108	0.19 (−0.11; 0.45)
B2 (mg)	1.30	0.73	1.34	0.49	1.20	0.77	1.31	0.73	0.611	0.07 (−0.21; 0.35)
B3 (mg)	17.91	11.41	18.13	7.33	12.51	9.94	15.23	7.73	0.114	0.23 (−0.06; 0.49)
B6 (mg)	1.53	0.77	1.62	0.60	1.21	0.97	1.40	0.58	0.167	0.20 (−0.10; 0.47)
B9 (mg)	230.71	139.85	225.64	71.86	200.35	201.11	225.76	135.68	0.545	0.09 (−0.21; 0.37)
C (mg)	64.50	76.01	89.04	71.29	70.33	117.25	105.97	103.91	0.869	0.02 (−0.27; 0.32)
D (µg)	1.79	1.95	2.32	1.60	1.92	1.86	2.38	1.89	0.783	0.04 (−0.24; 0.32)
E (mg)	7.13	5.26	8.41	6.06	4.96	4.07	5.64	3.30	0.016	0.35 (0.06; 0.59)

* *p*-value for the Mann–Whitney U test; significant if *p* ≤ 0.05. ** Absolute values < 0.15 indicate a negligible effect; values between 0.15 and <0.33 represent a small effect; values between 0.33 and <0.47 indicate a medium effect; and values ≥ 0.47 correspond to a large effect. BMI = body mass index; CI = confidence interval; IQR = interquartile range; OLP = oral lichen planus; SD = standard deviation.

**Table 4:** Sleep quality parameters in the Non-Erosive OLP, Erosive OLP, and Control groups.

	Non-Erosive OLP (n = 25)				Erosive OLP (n = 7)				Control (n = 31)				
Sleep Parameters	Median	IQR	Mean	SD	Median	IQR	Mean	SD	Median	IQR	Mean	SD	*p* *
PSQI (global)	9	7	8.32	4.356	9	5	10.29	3.147	6	6	7.23	4.417	0.126
PSQI (components)													
Subjective sleep quality	1	1	1.24	0.831	2	1	1.71	0.488	1	1	1.23	0.805	0.123
Sleep latency	2	1	1.52	1.046	2	2	1.71	1.113	1	2	1.45	1.121	0.830
Sleep duration	1	2	1.24	1.128	2	1	1.71	0.951	0	1	0.84	1.068	0.098
Habitual sleep efficiency	1	2	1.32	1.145	1	1	1.14	0.690	0	1	0.84	1.186	0.164
Sleep disturbances	2	1	1.76	0.723	2	0	1.71	0.756	1	1	1.42	0.502	0.067
Use of sleep medications	0 ^A^	0	0.48	1.046	1 ^B^	3	1.57	1.397	0 ^A,B^	2	0.68	1.194	0.040
Daytime dysfunction	1	1	0.76	0.723	1	1	0.71	0.756	1	1	0.77	0.669	0.968
ESS	7	7	7.80	4.349	6	5	6.57	3.690	6	6	5.87	4.602	0.277

* *p*-value for Kruskal–Wallis test; significant if *p* ≤ 0.05. Different superscript letters within a row indicate significant differences between groups (Dunn’s post hoc test). ESS = Epworth sleepiness scale; IQR = interquartile range; OLP = oral lichen planus; PSQI = Pittsburgh sleep quality index; SD = standard deviation.

**Table 5:** Spearman correlations between BMI, sleep quality parameters, cortisol, and melatonin.

Variables	BMI	EES	PSQI (Global)	Cortisol	Melatonin
BMI	1				
EES	−0.160	1			
PSQI (global)	0.046	−0.031	1		
Cortisol	−0.193	0.096	−0.315 *	1	
Melatonin	−0.068	0.112	−0.150	−0.021	1

* Significant correlation at 0.05 level. BMI = body mass index; PSQI = Pittsburgh sleep quality index; EES = Epworth sleepiness scale.

## Data Availability

The original contributions presented in this study are included in the article/Supplementary Material. Further inquiries can be directed to the corresponding author.

## Supplementary Materials

The following supporting information can be downloaded at: https://www.mdpi.com/article/10.3390/biomedicines14061275/s1, Supplementary Material: frequency of systemic diseases and medication use in the Oral Lichen Planus (OLP) and Control groups.

## Abbreviations

The following abbreviations are used in this manuscript:

BMI	Body mass index
ESS	Epworth sleepiness scale
IQR	Interquartile range
OLP	Oral lichen planus
PSQI	Pittsburgh sleep quality index
PUCRS	Pontifical Catholic University of Rio Grande do Sul
SD	Standard deviation
STROBE	Strengthening the Reporting of Observational Studies in Epidemiology
VAS	Visual analog scale

## References

[1] Song H.J., Kang K.H., Byun J.S., Kim D.Y. (2026). Oxidative stress and metabolic dysfunction in oral lichen planus pathogenesis. Anim. Cells Syst..

[2] González-Moles M.Á., Warnakulasuriya S., González-Ruiz I., González-Ruiz L., Ayén Á., Lenouvel D., Ruiz-Ávila I., Ramos-García P. (2021). Worldwide prevalence of oral lichen planus: A systematic review and meta-analysis. Oral Dis..

[3] Li K., He W., Hua H. (2022). Characteristics of the psychopathological status of oral lichen planus: A systematic review and meta-analysis. Aust. Dent. J..

[4] Manczyk B., Gołda J., Biniak A., Reszelewska K., Mazur B., Zając K., Bińczak P., Chomyszyn-Gajewska M., Oruba Z. (2019). Evaluation of depression, anxiety and stress levels in patients with oral lichen planus. J. Oral Sci..

[5] Vilar-Villanueva M., Gándara-Vila P., Blanco-Aguilera E., Otero-Rey E.M., Rodríguez-Lado L., García-García A., Blanco-Carrión A. (2019). Psychological disorders and quality of life in oral lichen planus patients and a control group. Oral Dis..

[6] Wiriyakijja P., Porter S., Fedele S., Hodgson T., McMillan R., Shephard M., Ni Riordain R. (2020). Validation of the HADS and PSS-10 and psychological status in patients with oral lichen planus. Oral Dis..

[7] Koray M., Dülger O., Ak G., Horasanli S., Uçok A., Tanyeri H., Badur S. (2003). The evaluation of anxiety and salivary cortisol levels in patients with oral lichen planus. Oral Dis..

[8] Pires A.L.P.V., Simoura J.A.D.S., Cerqueira J.D.M., Lima-Arsati Y.B.O., Arsati F., Dos Santos J.N., Freitas V.S. (2020). Relationship of psychological factors with salivary flow rate and cortisol levels in individuals with oral lichen planus: A case-control study. Oral Surg. Oral Med. Oral Pathol. Oral Radiol..

[9] Adamo D., Ruoppo E., Leuci S., Aria M., Amato M., Mignogna M.D. (2015). Sleep disturbances, anxiety and depression in patients with oral lichen planus: A case-control study. J. Eur. Acad. Dermatol. Venereol..

[10] Lopez-Jornet P., Cayuela C.A., Tvarijonaviciute A., Parra-Perez F., Escribano D., Ceron J. (2016). Oral lichen planus: Salival biomarkers cortisol, immunoglobulin A, adiponectin. J. Oral Pathol. Med..

[11] Adamo D., Calabria E., Coppola N., Lo Muzio L., Giuliani M., Bizzoca M.E., Azzi L., Croveri F., Colella G., Boschetti C.E. (2021). Psychological profile and unexpected pain in oral lichen planus: A case–control multicenter SIPMO study. Oral Dis..

[12] Gómez-Moreno G., Guardia J., Ferrera M.J., Cutando A., Reiter R.J. (2010). Melatonin in diseases of the oral cavity. Oral Dis..

[13] Poza J.J., Pujol M., Ortega-Albás J.J., Romero O., Insomnia Study Group of the Spanish Sleep Society (SES) (2022). Melatonin in sleep disorders. Neurología.

[14] Permuy M., López-Peña M., González-Cantalapiedra A., Muñoz F. (2017). Melatonin: A review of its potential functions and effects on dental diseases. Int. J. Mol. Sci..

[15] Kołodziejska R., Woźniak A., Bilski R., Wesołowski R., Kupczyk D., Porzych M., Wróblewska W., Pawluk H. (2025). Melatonin—A powerful antioxidant in neurodegenerative diseases. Antioxidants.

[16] Hardeland R. (2018). Melatonin and inflammation—Story of a double-edged blade. J. Pineal Res..

[17] Mańka S., Majewska E. (2016). Immunoregulatory action of melatonin. The mechanism of action and the effect on inflammatory cells. Postep. Hig. Med. Dosw..

[18] Skarlis C., Anagnostouli M. (2020). The role of melatonin in multiple sclerosis. Neurol. Sci..

[19] Chaiyarit P., Luengtrakoon K., Wannakasemsuk W., Vichitrananda V., Klanrit P., Hormdee D., Noisombut R. (2017). Biological functions of melatonin in relation to pathogenesis of oral lichen planus. Med. Hypotheses.

[20] Ying J., Xiang W., Qiu Y., Zeng X. (2020). Risk of metabolic syndrome in patients with lichen planus: A systematic review and meta-analysis. PLoS ONE.

[21] Herbert D., Franz S., Popkova Y., Anderegg U., Schiller J., Schwede K., Lorz A., Simon J.C., Saalbach A. (2018). High-fat diet exacerbates early psoriatic skin inflammation independent of obesity: Saturated fatty acids as key players. J. Investig. Dermatol..

[22] Zhang J., Zhou G. (2012). Green tea consumption: An alternative approach to managing oral lichen planus. Inflamm. Res..

[23] van der Meij E.H., van der Waal I. (2003). Lack of clinicopathologic correlation in the diagnosis of oral lichen planus based on the presently available diagnostic criteria and suggestions for modifications. J. Oral Pathol. Med..

[24] Cutando A., Gómez-Moreno G., Villalba J., Ferrera M.J., Escames G., Acuña-Castroviejo D. (2003). Relationship between salivary melatonin levels and periodontal status in diabetic patients. J. Pineal Res..

[25] Chang W.P., Lin C.C. (2017). Relationships of salivary cortisol and melatonin rhythms to sleep quality, emotion, and fatigue levels in patients with newly diagnosed lung cancer. Eur. J. Oncol. Nurs..

[26] Thongprasom K., Luangjarmekorn L., Sererat T., Taweesap W. (1992). Relative efficacy of fluocinolone acetonide compared with triamcinolone acetonide in treatment of oral lichen planus. J. Oral Pathol. Med..

[27] von Elm E., Altman D.G., Egger M., Pocock S.J., Gøtzsche P.C., Vandenbroucke J.P., STROBE Initiative (2014). The strengthening the reporting of observational studies in epidemiology (STROBE) statement: Guidelines for reporting observational studies. Int. J. Surg..

[28] Bertolazi A.N., Fagondes S.C., Hoff L.S., Dartora E.G., Miozzo I.C., de Barba M.E., Barreto S.S. (2011). Validation of the Brazilian Portuguese version of the Pittsburgh Sleep Quality Index. Sleep Med..

[29] Bertolazi A.N., Fagondes S.C., Hoff L.S., Pedro V.D., Barreto S.S.M., Johns M.W. (2009). Portuguese-language version of the Epworth sleepiness scale: Validation for use in Brazil. J. Bras. Pneumol..

[30] Akkaoui M.A., Palagini L., Geoffroy P.A. (2023). Sleep immune cross talk and insomnia. Adv. Exp. Med. Biol..

[31] Murakami Y., Machino M., Fujisawa S. (2012). *Porphyromonas gingivalis* fimbria-induced expression of inflammatory cytokines and cyclooxygenase-2 in mouse macrophages and its inhibition by the bioactive compounds fibronectin and melatonin. ISRN Dent..

[32] Luengtrakoon K., Wannakasemsuk W., Vichitrananda V., Klanrit P., Hormdee D., Noisombut R., Chaiyarit P. (2017). Increased melatonin in oral mucosal tissue of oral lichen planus (OLP) patients: A possible link between melatonin and its role in oral mucosal inflammation. Arch. Oral Biol..

[33] Jose S., Mukundan J.V., Johny J., Tom A., Mohan S.P., Sreenivasan A. (2019). Estimation of serum cortisol levels in oral lichen planus patients with electrochemiluminescence. J. Pharm. Bioallied Sci..

[34] Girardi C., Luz C., Cherubini K., de Figueiredo M.A., Nunes M.L., Salum F.G. (2019). Salivary cortisol and dehydroepiandrosterone (DHEA) levels, psychological factors in patients with oral lichen planus. Arch. Oral Biol..

[35] Glavina A., Lugović-Mihić L., Martinović D., Cigić L., Tandara L., Lukenda M., Biočina-Lukenda D., Šupe-Domić D. (2023). Association between salivary cortisol and α-amylase with the psychological profile of patients with oral lichen planus and burning mouth syndrome: A case–control study. Biomedicines.

[36] Lopez-Jornet P., Zavattaro E., Mozaffari H.R., Ramezani M., Sadeghi M. (2019). Evaluation of the salivary level of cortisol in patients with oral lichen planus: A meta-analysis. Medicina.

[37] Manzel A., Muller D.N., Hafler D.A., Erdman S.E., Linker R.A., Kleinewietfeld M. (2014). Role of “western diet” in inflammatory autoimmune diseases. Curr. Allergy Asthma Rep..

[38] Mazzucca C.B., Raineri D., Cappellano G., Chiocchetti A. (2021). How to tackle the relationship between autoimmune diseases and diet: Well begun is half-done. Nutrients.

[39] López-Jornet P., Camacho-Alonso F., Rodríguez-Martnes M.A. (2012). Alterations in serum lipid profile patterns in oral lichen planus: A cross-sectional study. Am. J. Clin. Dermatol..

[40] Ozbagcivan O., Akarsu S., Semiz F., Fetil E. (2020). Comparison of serum lipid parameters between patients with classic cutaneous lichen planus and oral lichen planus. Clin. Oral Investig..

[41] Okunogbe A., Nugent R., Spencer G., Ralston J., Wilding J. (2021). Economic impacts of overweight and obesity: Current and future estimates for eight countries. BMJ Glob. Health.

[42] Li K.Y., Li C.L., Hua H., Song Z.F. (2023). Potential relationship of dyslipidemia with dietary patterns in oral lichen planus patients-a case-control study. J. Dent. Sci..

[43] Gholizadeh N., Mehdipour M., Najafi S., Bahramian A., Garjani S., Khoeini Poorfar H. (2014). Evaluation of the serum zinc level in erosive and non-erosive oral lichen planus. J. Dent..

[44] Wessels I., Maywald M., Rink L. (2017). Zinc as a gatekeeper of immune function. Nutrients.

[45] Zhu Z., He Y., Wu F., Zhao L., Wu C., Lu Y., Zang J., Wang Z., Sun J., Huang J. (2020). The associations of dietary iron, zinc and magnesium with metabolic syndrome in China’s mega cities. Nutrients.

[46] Gholizadeh N., Sheykhbahaei N. (2021). Micronutrients profile in oral lichen planus: A review literature. Biol. Trace Elem. Res..

[47] Bhattacharya P.T., Misra S.R., Hussain M. (2016). Nutritional aspects of essential trace elements in oral health and disease: An extensive review. Scientifica.

[48] Wang J., Yang J., Wang C., Zhao Z., Fan Y. (2021). Systematic review and meta-analysis of oxidative stress and antioxidant markers in oral lichen planus. Oxid. Med. Cell Longev..

[49] National Academies of Sciences, Engineering, and Medicine (2006). Dietary Reference Intakes: The Essential Guide to Nutrient Requirements.

[50] Sinha R., Patterson B.H., Mangels A.R., Levander O.A., Gibson T., Taylor P.R., Block G. (1993). Determinants of plasma vitamin E in healthy males. Cancer Epidemiol. Biomark. Prev..

[51] Yang J., Wang A., Shang L., Sun C., Jia X., Hou L., Xu R., Wang X. (2022). Study on the association between dietary habits, patterns and frailty of the elderly: A cross-sectional survey from communities in China. Clin. Interv. Aging.

[52] Román G.C., Jackson R.E., Gadhia R., Román A.N., Reis J. (2019). Mediterranean diet: The role of long-chain ω-3 fatty acids in fish; polyphenols in fruits, vegetables, cereals, coffee, tea, cacao and wine; probiotics and vitamins in prevention of stroke, age-related cognitive decline, and Alzheimer disease. Rev. Neurol..

[53] Salehi B., Lopez-Jornet P., Pons-Fuster López E., Calina D., Sharifi-Rad M., Ramírez-Alarcón K., Forman K., Fernández M., Martorell M., Setzer W.N. (2019). Plant-derived bioactives in oral mucosal lesions: A key emphasis to curcumin, lycopene, chamomile, aloe vera, green tea and coffee properties. Biomolecules.

[54] Sheth C.C., López-Pedrajas R.M., Jovani-Sancho M.D.M., González-Martínez R., Veses V. (2018). Modulation of salivary cytokines in response to alcohol, tobacco and caffeine consumption: A pilot study. Sci. Rep..

[55] Rhodus N.L., Cheng B., Bowles W., Myers S., Miller L., Ondrey F. (2006). Proinflammatory cytokine levels in saliva before and after treatment of (erosive) oral lichen planus with dexamethasone. Oral Dis..

[56] Popa C., Sciuca A.M., Onofrei B.-A., Toader S., Condurache Hritcu O.M., Boțoc Colac C., Porumb Andrese E., Brănișteanu D.E., Toader M.P. (2024). Integrative approaches for the diagnosis and management of erosive oral lichen planus. Diagnostics.

[57] Onen S.H., Onen F. (2018). Chronic medical conditions and sleep in the older adult. Sleep Med. Clin..

[58] Frohnhofen H. (2018). Pain and sleep: A bidirectional relationship. Z. Gerontol. Geriatr..

[59] Perneger T.V. (1998). What’s wrong with Bonferroni adjustments. BMJ.

[60] Boulesteix A.L., Hoffmann S. (2024). To adjust or not to adjust: It is not the tests performed that count, but how they are reported and interpreted. BMJ Med..

[61] Ribeiro A.C., Sávio K.E.O., Rodrigues M.L.C.F., Costa T.H.M., Schmitz B.A.S. (2006). Validation of a food frequency questionnaire for the adult population. Rev. Nutr..

[62] Salvador Castell G., Serra-Majem L., Ribas-Barba L. (2015). What and how much do we eat? 24-hour dietary recall method. Nutr. Hosp..

[63] Czerninski R., Zadik Y., Kartin-Gabbay T., Zini A., Touger-Decker R. (2014). Dietary alterations in patients with oral vesiculoulcerative diseases. Oral Surg. Oral Med. Oral Pathol. Oral Radiol..

