# Supplementary material for: Association of Sleep Quality, Nutritional Factors, and Salivary Melatonin and Cortisol Levels with Oral Lichen Planus: A Case–Control Study
Source: Biomedicines. 2026 Jun 3;14(6):1275. doi: 10.3390/biomedicines14061275 (PMC13296431; doi:10.3390/biomedicines14061275)
Supplement: Supplementary file 1 [file biomedicines-14-01275-s001.zip › biomedicines-4304202-supplementary.pdf]

Supplementary material: frequency of systemic diseases and medication use in the Oral Lichen Planus (OLP) and Control groups

**Systemic disease**	**OLP** (n=32)		**Control** (n=31)		***P***
	**n**	**%**	**n**	**%**	
Systemic arterial hypertension	20	62.50	16	51.61	0.382*
Hypercholesterolemia	9	28.13	11	35.48	0.530*
Depression	9	28.13	7	22.58	0.613*
Gastroesophageal reflux/gastritis	7	21.88	6	19.35	0.805*
Hypothyroidism	5	15.63	3	9.68	0.708**
Diabetes	6	18.75	2	6.45	0.256**
Osteoporosis/osteopenia	1	3.13	6	19.35	0.053**
Cardiopathy	2	6.25	2	6.45	1.000**
Asthma	0	0.00	1	3.23	0.492**
**Drugs Used**					
Statins	9	28.13	11	35.48	0.530*
Angiotensin II receptor blockers	13	40.63	6	19.35	0.659*
Beta-adrenergic receptor blockers	8	25.00	6	19.35	0.590*
Calcium channel blockers	1	3.13	4	12.90	0.196**
Diuretics	10	31.25	5	16.13	0.159*
Antidepressants	9	28.13	7	22.58	0.613*
Antidiabetics (metformin/glibenclamide)	7	21.88	2	6.45	0.148**
Levothyroxine	5	15.63	3	9.68	0.708**
Proton pump inhibitors	7	21.88	5	16.13	0.561*
Anticoagulants	1	3.13	0	0.00	1.000**
Platelet antiaggregants	5	15.63	6	19.35	0.697*
Oral bisphosphonates	1	3.13	6	19.35	0.053**
Coronary vasodilators	1	3.13	3	9.68	0.355**
Bronchodilators	0	0.00	1	3.23	0.492**

*Chi-square test; **Fisher's exact test; significant if P≤0.05
